# Harvest‐induced evolution and effective population size

**DOI:** 10.1111/eva.12373

**Published:** 2016-04-08

**Authors:** Anna Kuparinen, Jeffrey A. Hutchings, Robin S. Waples

**Affiliations:** ^1^ Department of Environmental SciencesUniversity of HelsinkiHelsinkiFinland; ^2^ Department of BiologyDalhousie UniversityHalifaxNSCanada; ^3^ Department of BiosciencesCentre For Ecological and Evolutionary SynthesisUniversity of OsloOsloNorway; ^4^ Department of Natural SciencesUniversity of AgderKristiansandNorway; ^5^ National Marine Fisheries ServiceNational Oceanic and Atmospheric AdministrationNorthwest Fisheries Science CenterSeattleWAUSA

**Keywords:** contemporary evolution, fisheries management, life history evolution, population genetics – empirical, wildlife management

## Abstract

Much has been written about fishery‐induced evolution (FIE) in exploited species, but relatively little attention has been paid to the consequences for one of the most important parameters in evolutionary biology—effective population size (*N*_e_). We use a combination of simulations of Atlantic cod populations experiencing harvest, artificial manipulation of cod life tables, and analytical methods to explore how adding harvest to natural mortality affects *N*_e_, census size (*N*), and the ratio *N*_e_/*N*. We show that harvest‐mediated reductions in *N*_e_ are due entirely to reductions in recruitment, because increasing adult mortality actually increases the *N*_e_/*N* ratio. This means that proportional reductions in abundance caused by harvest represent an upper limit to the proportional reductions in *N*_e_, and that in some cases *N*_e_ can even increase with increased harvest. This result is a quite general consequence of increased adult mortality and does not depend on harvest selectivity or FIE, although both of these influence the results in a quantitative way. In scenarios that allowed evolution, *N*_e_ recovered quickly after harvest ended and remained higher than in the preharvest population for well over a century, which indicates that evolution can help provide a long‐term buffer against loss of genetic variability.

## Introduction

Increasingly in recent decades, humans have created a global experiment by subjecting natural populations to harvest at rates that equal or exceed the rate of natural mortality (Darimont et al. 2009). Some short‐term consequences of harvest can be deduced from first principles. The additional harvest‐induced mortality will truncate the age structure of the population because fewer individuals live to old age. Moreover, this additional mortality is often positively correlated with size, due to harvesting regulations and trophy hunting (Coltman et al. 2003; Allendorf and Hard 2009). Size in turn is correlated with age in species with indeterminate growth, such that the effect of age‐structure truncation will be exacerbated.

These short‐term demographic consequences can be expected to elicit evolutionary responses in species with the genetic capability to do so. Species with low rates of natural mortality as adults generally mature at older ages, because investing limited energy into growth rather than early maturity means that they will be larger when they reach maturity (and hence have higher fecundity and potentially higher mating success), and they can expect to reap the benefits of higher fecundity for many years because mortality is low. If adult mortality is sharply increased, perhaps by a factor of 2 or more (Mertz and Myers 1998; Law 2007), individuals that delay reproduction no longer can expect to enjoy many seasons of high reproductive success, so relative fitness of that phenotype declines. The result is evolutionary pressure to mature at an earlier age and smaller size, to ensure at least some opportunities for reproduction before death. Precisely predicting evolutionary responses to harvest is difficult because changes in a population's vital rates can affect density dependence, particularly at juvenile life stages, as well as biotic interactions with other species (Polacheck et al. 2004; Howell et al. 2013; Kuparinen et al. 2014a). Nevertheless, numerous studies have estimated empirical rates of phenotypic change in harvested species that are in line with expectations from fisheries‐induced evolution (FIE) (Hutchings and Baum 2005; Sharpe and Hendry 2009; Devine et al. 2012; Audzijonyte et al. 2013; Kendall et al. 2014).

Over the past decades, numerous studies have focused on FIE, to understand its mechanisms and to project its ecological consequences. However, this literature has largely ignored influences of FIE on effective population size (*N*_e_). This is an important gap because *N*_*e*_ can influence virtually all evolutionary processes. Effective size determines not only the rates of inbreeding, allele frequency change, and loss of genetic variability in a population, but also the efficiency of natural selection (and hence the balance between random and directed evolutionary processes; see Edeline et al. 2007; Lanfear et al. 2014). *N*_*e*_ and the ratio of *N*_e_ to census size (*N*) are sensitive to population demography (Felsenstein 1971; Nunney 1993), so direct, short‐term effects of harvest and longer‐term evolutionary changes to a population's vital rates can both be expected to change *N*_e_ and *N*_e_/*N*.

One notable exception to the above gap regarding effective population size is the study by Marty et al. (2015), who showed that considering random effects associated with FIE is important, particularly when evaluating potential for evolutionary recovery after fishing is relaxed. They showed that, in many circumstances, random factors related to *N*_e_ can be more important than FIE in eroding additive genetic variance, which provides evolutionary resilience to a population. Marty et al. (2015) simulated both neutral and adaptive genes and estimated *N*_e_ from neutral genes by tracking the rate of change in allele frequency over time (the temporal method; Waples 1989). They took samples every 20 years and converted this time interval into elapsed generations based on calculations of generation length (*T*) from the simulated demographies. This approach should be sufficient to provide rough estimates of *N*_e_. However, the standard temporal method they used assumes discrete generations and is not ideally suited for iteroparous species with overlapping generations—exactly the type of species most likely to experience FIE (Hutchings and Fraser 2008). Based on the range of generation lengths in their modeled populations (*T *=* *7.5–12.1 years; Marty et al. 2015), each 20‐year period for estimating *N*_e_ encompassed only 1.7–2.7 generations, which is not enough to eliminate age‐structure bias in ${\hat{N}}_{e}$ in the temporal method (Waples and Yokota 2007). Furthermore, the resulting estimates apply to a harmonic mean *N*_e_ over the period between samples and hence are difficult to relate to specific points in time.

Here, we take a different approach and calculate *N*_e_ directly from vital rates for simulated populations of Atlantic cod that experience various harvest scenarios previously modeled, for example, by Kuparinen et al. (2014a). We use a method for calculating *N*_e_ (AgeNe; Waples et al. 2011) that is designed for use with iteroparous, age‐structured species and which can estimate effective size for individual cohorts. We consider both *N*_e_ and the ratio *N*_e_/*N* (with *N* defined as the number of mature adults) because the latter allows us to disentangle the effects of changes in vital rates that affect the *N*_e_/*N* ratio from effects on abundance, which can reduce *N*_e_ even if *N*_e_/*N* is not reduced. To explore generality of our results, we supplement the simulations with analytical results and artificial manipulation of another life table for Atlantic cod.

## Methods

Table 1 lists notation used in this study. Our analyses used two different life tables for Newfoundland's Northern cod, which we refer to as cod life table #1 and cod life table #2. These life tables are both based on empirical data, but for different areas and time periods with different histories of exploitation. Life table #1 was used to parameterize the simulations that evaluated demographic and evolutionary responses to fishing. These simulations included density dependence, again based on empirical data. To explore generality of our simulation results, we artificially manipulated cod life table #2 by increasing adult mortality. These analyses were purely demographic and did not consider evolution or density dependence. More details about each type of analysis are provided below.

**Table 1:** Notation used in this study

*N*_T_	Total population size, including juveniles
*N*	Adult population size (all mature individuals)
*N*_e_	Effective population size per generation
*α*	Youngest age at which reproduction can occur
*ω*	Maximum age
*N*_1_	Number of newborn offspring produced each year.
*N*_*α*_	Number of offspring produced each year that survive to age at first reproduction, at which point they are known as *recruits*
*b*_*x*_	Mean number of offspring per year produced by an individual of age *x* that survive to age of recruitment
*s*_*x*_	Probability of survival from age *x* to age *x *+* *1
*d*_*x*_	=1−*s*_*x*_ = probability of dying between age *x* and age *x *+* *1
*l*_*x*_	Cumulative survival through age *x*
*T*	Generation length = average age of parents of a newborn cohort
*V*_*k*•_	Lifetime variance in reproductive success among individuals in a single cohort
*V*_*x*_	Variance in number of offspring produced by same‐age, same‐sex individuals in one time period
*ϕ*_*x*_	*V*_*x*_/*b*_*x*_ = ratio of the variance to mean number of offspring produced in one time period by individuals of age *x*
*F*	Instantaneous rate of fishing mortality (annual mortality = 1−*e* ^(−*F*)^)
*L*(*t*)	Length at age *t*
*L*_*∞*_	Asymptotic length
*k*	von Bertalanffy intrinsic growth coefficient

### Simulation of cod dynamics and construction of life tables

To investigate the impacts of fishing and FIE on *N*_e_, we constructed cod life table #1 at different phases of exploitation and fisheries‐induced life‐history evolution. To this end, we simulated cod dynamics using an individual‐based modeling approach that integrates quantitative genetics, life‐history evolution, and ecological dynamics of the population. Individual life histories are described through von Bertalanffy growth trajectories (von Bertalanffy 1938), *L(t) *= *L*_*∞*_−(*L*_*∞*_−*L*_0_)*e* ^−*kt*^, where *L*_0_ and *L(t)* are length at ages 0 and *t*,* L*_*∞*_ is asymptotic body length, and *k* is the intrinsic growth coefficient describing the speed at which *L*_*∞*_ is reached.

Genetic contributions to life histories were described through additive effects of 10 diploid loci (coded 0 or 1), to mimic the fact that quantitative traits are typically coded by many loci with small additive effects (Roff 2002). The sum of allelic values (ranging between 0 and 20) was coupled with a small amount of environmental variation (drawn from a normal distribution with mean = 0, SD = 3.5) to yield realistic heritabilities of ~0.2–0.3 for life‐history traits (Mousseau and Roff 1987; Carlson and Seamons 2008; but see also Postma 2014) and translated linearly into the value of *L*_*∞*_. The correlations between *k* and *L*_*∞*_ and between *L*_*∞*_ and the length at maturation are well‐established life‐history relationships (Charnov 1993; Charnov et al. 2013), so the value of *k* and the length at maturation could be estimated based on *L*_*∞*_. Empirical bases for the growth parameters and their relationships were obtained from growth trajectories estimated from otoliths collected in a landlocked cod population in Baffin Island, northern Canada. Cod life histories in this population are similar to marine cod populations in northern latitudes, and the population is unexploited and, therefore, reflects natural phenotypic diversity of cod life histories. The empirically observed range of *L*_*∞*_ was 30–130 cm, and *k* could be estimated through regression as log(*k*) = 0.609‐0.0139 × *L*_*∞*_ (with residual standard error of 0.305) (Kuparinen et al. 2012). *L*_*0*_ was set to 4 cm for each growth trajectory. The age–length relationship was estimated from the same cod data as *weight* = 3.52 × 10^6 ^× *length* ^3.19^.

Population dynamics were simulated through time such that at each time step (year) the processes of natural mortality, growth, maturation, and reproduction were modeled on an individual basis. Demographic stochasticity was accounted for by drawing appropriate random numbers to describe the outcome of each process. Baseline instantaneous natural mortality was assumed to be 0.12, to which a survival cost of reproduction of 0.1 was added for mature individuals; these values provide the closest match between the empirically observed cod growth trajectories and those predicted by the model (Kuparinen et al. 2012). Growth occurred such that at each time step an individual progressed along its von Bertalanffy growth trajectory according to a time increment Δ*t* = *e* ^15–17.69*c*^ (1 + *e* ^15–17.69*c*^)^−1^, where *c* is the ratio of population biomass to carrying capacity (*K*). In a sparse population, Δ*t* was approximately 1, corresponding to 1 year increment in simulation time, whereas in a dense population the progress is slower. Maturation was assumed to occur at a body length 66% of *L*_*∞*_ (Jensen 1997), and maximum age was set to 25 years.

At each time step, all mature individuals reproduced, such that for each mature female a mature male was assigned randomly (no sexual selection was assumed). Alleles were passed from parents to juveniles stochastically through Mendelian inheritance. Egg production was predicted through *eggs *= {0.48 × [(*female weight* + 0.37]/1.45) + 0.12} × 10^6^, as estimated for Northern cod in the 1960s (Hutchings 2005). At that time, abundance of the Northern cod stock was assumed to be at about 40% of its carrying capacity. Density dependence of juvenile production was assumed to be compensatory, such that the above egg production was scaled up or down according to the abundance‐specific relative fecundity estimates reported in Kuparinen et al. (2014b). Survival from egg to a 3‐year‐old recruit was set to 1.13 × 10^−6^ (Hutchings 2005). For further details of the model and its parameterization, see Kuparinen et al. (2012, 2014b).

Dynamics of preadapted cod populations were simulated first for 100 years in equilibrium conditions, followed by a 50‐year period of fishing and a 150‐year period of recovery in the absence of fishing. Simulations were repeated with and without life‐history evolution. In nonevolving simulations, juvenile alleles were drawn from a parental pool recorded during equilibrium conditions. We considered three alternative fishing pressures (*F *=* *0.15, *F *=* *0.20, and *F *=* *0.25, where *F* is instantaneous fishing mortality expressed as a fraction of total biomass) and two fishing selectivity scenarios (logistic typical for trawl, and no size selectivity). These fishing intensities are well within the range of population‐specific target fishing mortality levels for Atlantic cod (F_MSY_: 0.18–0.40; www.ices.dk). However, we needed to model levels that were sustainable over five decades and left a large enough population to allow calculation of age‐specific vital rates. At each time step throughout the simulations, we recorded age‐specific survival (*s*_*x*_), fecundity (*b*_*x*_), and the proportion of mature individuals, as well as total annual recruit production. Life tables for the simulated populations were then compiled by averaging across replicates at specific years representing the period of equilibrium (year 100); early fishing (years 110, 130); late fishing (year 150), by which point fisheries‐induced evolution had occurred in evolving populations; initial recovery following the end of fishing (years 160, 180); mid recovery (year 220); and late recovery, by which time biomass had rebuilt back to equilibrium levels (year 300).

### Census size and effective population size

#### Census size

In a stable, age‐structured population, total population size (*N*_T_) depends on two parameters: the number of newborns each year (*N*_1_) and cumulative survivorship over time (*l*_*x*_), calculated through the maximum age (*ω*). Adult population size (*N*) can be obtained by replacing newborns with recruits (*N*_*α*_ = the number of offspring that survive to age at maturity, *α*), defining *l*_*α*_ to be 1, and taking the sum across the years of the adult life span (*α* to *ω*):

(1) $$N=N_{\alpha }\sum_{x=\alpha }^{\omega }l_{x}$$

Because age at maturity varies in cod (Table 2), in calculating adult *N* from eqn (1) we used *α* = 3 (the minimum age any individuals matured in our study) and adjusted Σ*l*_*x*_ to account for the fraction mature at each age.

**Table 2:** Fraction of individuals that survive to age 3–10 that are sexually mature, at three time points in simulations with (*E*) and without (*NE*) evolution

Age	Year 100 Equilibrium	Year 150		Year 300		150E/Eq	150NE/Eq
		*NE*	*E*	*NE*	*E*		
1	0	0	0	0	0	–	–
2	0	0	0	0	0	–	–
3	0.005	0.047	0.068	0.006	0.017	14.0	9.7
4	0.039	0.180	0.247	0.043	0.088	6.4	4.7
5	0.123	0.379	0.425	0.132	0.227	3.5	3.1
6	0.249	0.492	0.624	0.264	0.397	2.5	2.0
7	0.396	0.662	0.732	0.413	0.555	1.8	1.7
8	0.531	0.775	0.837	0.553	0.683	1.6	1.5
9	0.650	0.812	0.858	0.666	0.784	1.3	1.2
10	0.739	0.839	0.930	0.750	0.850	1.3	1.1

Year 100 is the end of the equilibrium period before fishing; year 150 is the end of fishing and beginning of recovery, and year 300 is late recovery. The last two columns on the right show the ratio of results for year 150 with (and without) evolution to year 100 equilibrium. These data are for selective fishing with *F *=* *0.2 and are based on cod life table #1.

If adult mortality is constant at the rate *d* per year, then it can be shown that Σ*l*_*x*_ = 1/*d* and

(2) $$N=N_{\alpha }/d$$

This result is exact for a species with an arbitrarily long life span (Waples, in review) and is a good approximation for a long‐lived species like Atlantic cod.

#### Effective population size

We used the software AgeNe (Waples et al. 2011) to calculate *N*_e_ and *N*_e_/*N* at specific time steps, based on population vital rates calculated as described above. AgeNe uses Hill's (1972) general formula for calculating *N*_e_ for species with overlapping generations but retains the direct link to population vital rates provided by the method of Felsenstein (1971):

(3) $$N_{e}=\frac{4N_{\alpha }T}{V_{k∙}+2},$$

where (in our notation) *N*_*α*_ is the number of offspring produced each time period that survive to become recruits, *V*_*k*•_ is lifetime variance in reproductive success of the *N*_*α*_ recruits in a cohort, and *T* is generation length. AgeNe calculates lifetime *V*_*k*•_ from a population's vital rates by grouping individuals by age at death (see Waples et al. 2011). *N*_*α*_ is a scaling parameter; *N* and *N*_e_ both increase linearly with *N*_*α*_, but the ratio *N*_e_/*N* does not depend on *N*_*α*_. Similarly, mortality that occurs before maturity affects both *N* and *N*_e_ in the same way but not the ratio *N*_e_/*N*. AgeNe automatically rescales relative age‐specific fecundities to produce a stable population, and it also follows the Felsenstein and Hill models in assuming stable age structure and independence of survival and reproduction across time periods.

One final piece of information is required to calculate *N*_e_: *ϕ*_*x*_ = *V*_*x*_/*b*_*x*_ = the ratio of variance to mean reproductive success in one season for individuals of age *x*. If reproductive success of same‐age, same‐sex individuals is random, then each age and sex behaves like a mini Wright–Fisher ideal population, and *ϕ *≈ 1. Values of *ϕ *> 1 therefore represent overdispersed variance in reproductive success. To parameterize this part of the model, we drew on experimental data for three captive populations in which parentage analysis was used to assign offspring (fertilized eggs) to potential parents (see Supporting Information for details). Table S1 shows an example of age‐specific vital rates for the simulated population at equilibrium before harvest (year 100), after harvest (year 150), and late recovery (year 300).

AgeNe is based on discrete‐time life tables and requires the user to specify a maximum age, *ω*. In each scenario, we chose *ω* as the oldest age (≤25) for which both age‐specific survival and fecundity data were available; this was limited by low numbers of individuals that survived to advanced age, particularly in populations whose abundance declined sharply due to harvest. Resulting life tables for representative scenarios can be found in Table S1. At each time period in each scenario, the mean number of recruits produced per year was used as the value for *N*_*α*_ in the AgeNe calculations. Because vital rates in the simulations were only tracked for females, we used the same estimates for males in the AgeNe analyses.

#### Artificial manipulation of a life table for cod

Finally, to further explore generality of the above results, we artificially manipulated another life table for Atlantic cod (cod life table #2), based on data from Hutchings (2011) as modified by Waples et al. (2013). In this population, cod do not mature until age 7 and have maximum age *ω* = 20, constant annual adult survival at *s*_*x*_ = 0.82, and fecundity that increases with age (Table S2). We created variations of this life table by allowing annual adult survival to drop to 0.72, 0.62, and 0.52 to reflect an increasing but uniform harvest rate that changed annual adult mortality to *d *=* *0.28, 0.38, and 0.48, respectively. In the original population, the fraction of adults reaching age 7 that were still alive at age 20 was 0.82^13^ = 0.076. Therefore, in the three artificial populations we truncated the life table at *ω* = the first age when cumulative survival from age 7 dropped below 0.076. In the variations with *s*_*x*_ **= 0.72, 0.62, and 0.52, this resulted in *ω* = 16, 14, and 12, respectively. We considered three general scenarios, each with variable adult survival: (i) fecundity is constant and *ϕ* is fixed at 3, which is roughly the value we estimated for age 15 in a pristine population; (ii) relative fecundity increases with age in the same relative proportions as in the original life table, and *ϕ* is fixed at 3; and (iii) fecundity and *ϕ* both increase with age, with the increase in *ϕ* following the same schedule we used for the simulated populations, except we started with *ϕ *= 1 at age 7 rather than age 3. These scenarios did not consider either evolution of earlier age at maturity or potential density‐dependent effects of increasing adult mortality on population dynamics, so *N*_1_ was assumed to remain constant. Nonetheless, they provide insights into consequences for age structure and *N*_e_/*N* associated with changes in adult mortality.

## Results

### Simulations of harvest and recovery

**Figure 1:**
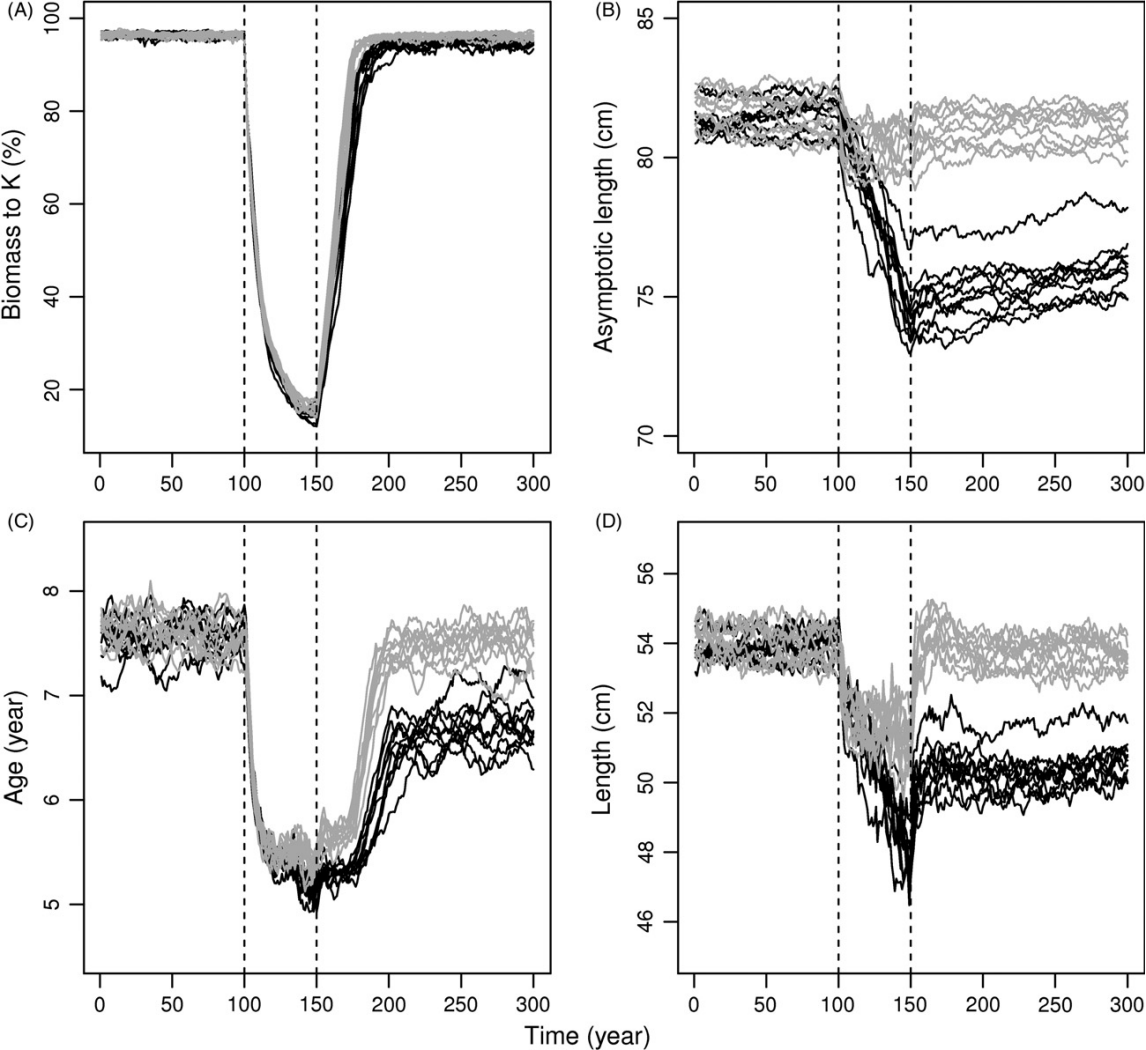
The temporal development of cod population biomass (A), asymptotic body length (B), age at maturity (C), and size at maturity (D) in ten replicated simulation runs, each described by a solid line. Evolving simulations are drawn with black and nonevolving simulations with gray. The beginning and the end of fishing period are denoted with vertical dashed lines.

Fishing led to steep declines of cod population biomass, such that by the end of the fishing period the biomass had dropped below 20% of population carrying capacity (Fig. 1A, with selective harvest). Owing to selective removal of large individuals, fisheries‐induced evolution caused asymptotic body length to decline across the fishing period by about 7 cm (Fig. 1B). Similar declines were also seen in the age and size at maturation, but the difference between evolving and nonevolving scenarios was less pronounced, as relaxed density‐dependent competition accelerated growth and allowed fish to reach maturity earlier (Fig. 1C,D). After fishing ceased, biomass recovered rapidly to the prefishing level, but evolutionary recovery of the life‐history traits was much slower, and clear differences in asymptotic length and age and size at maturity could still be seen at the end of the simulations.

#### Changes in demographic parameters

**Figure 2:**
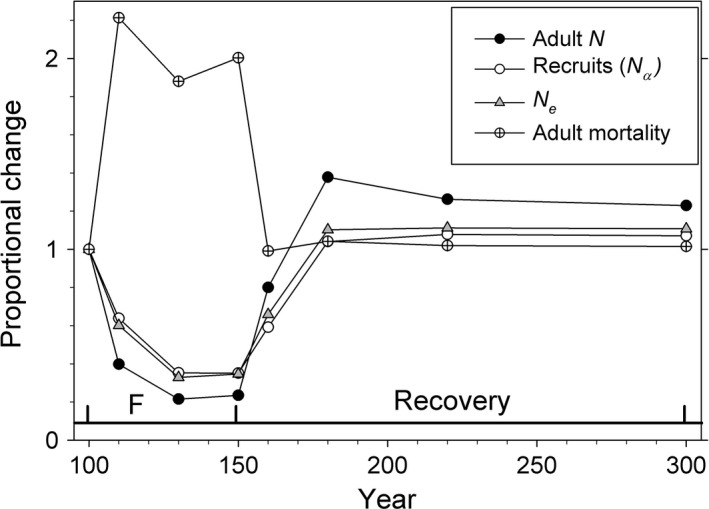
Proportional change in key demographic parameters over the course of the simulations. Results are for selective fishing at *F *=* *0.2, with evolution. Time periods indicate the end of equilibrium and start of fishing (year 100), end of fishing and beginning of recovery (year 150), and late recovery (year 300).

Figure 2 shows how key demographic parameters changed over the course of a typical simulation (selective harvest at *F *=* *0.2, with evolution). Adding harvest on top of natural mortality roughly doubled the total adult mortality experienced by the population. As a consequence, adult *N* declined sharply during harvest before rapidly returning to its original status after harvest finished. The number of recruits (*N*_*α*_) also declined sharply during harvest, but not as much as did *N*. Changes in annual survival between the equilibrium population and the end of fishing (year 150) are shown for several scenarios in Figure S1.

The purely demographic consequences for age at maturity of harvesting at this level can be seen by focusing on results where evolution was not allowed (Table 2). By the end of fishing at year 150, the fraction that were mature at young ages (3–5) was 3–10 times higher than in the equilibrium population before fishing (year 100), and the first age at which 50% of the population was mature had been reduced from 8 to 6. This occurred because increased adult mortality reduced overall abundance, and juvenile growth was enhanced owing to reduced density‐dependent competition, allowing fish to reach body size at which they matured (66% of *L*_*∞*_) at a younger age. By year 300 (late recovery), age at maturity in scenarios without evolution had largely returned to the preharvest equilibrium pattern (Table 2).

#### Patterns of change in *N*,* N*_e_ and *N*_e_/*N*

**Figure 3:**
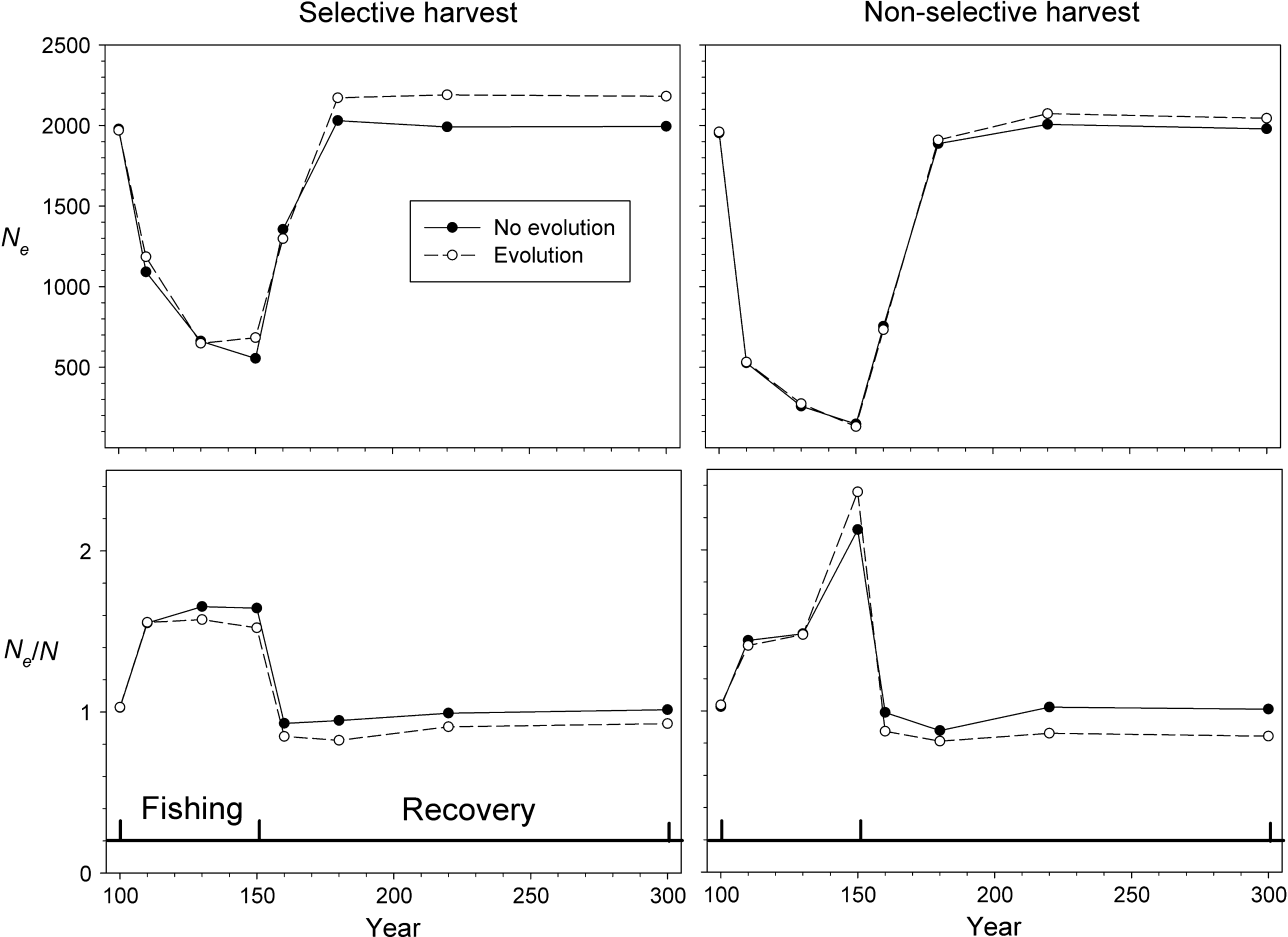
Changes in *N*_e_ and *N*_e_/*N* over the course of simulations with *F *=* *0.2. Results are shown for scenarios with selective harvest (left panels) and nonselective harvest (right panels), and that do (open circles) and do not (filled circles) allow evolution of life‐history traits.

**Figure 4:**
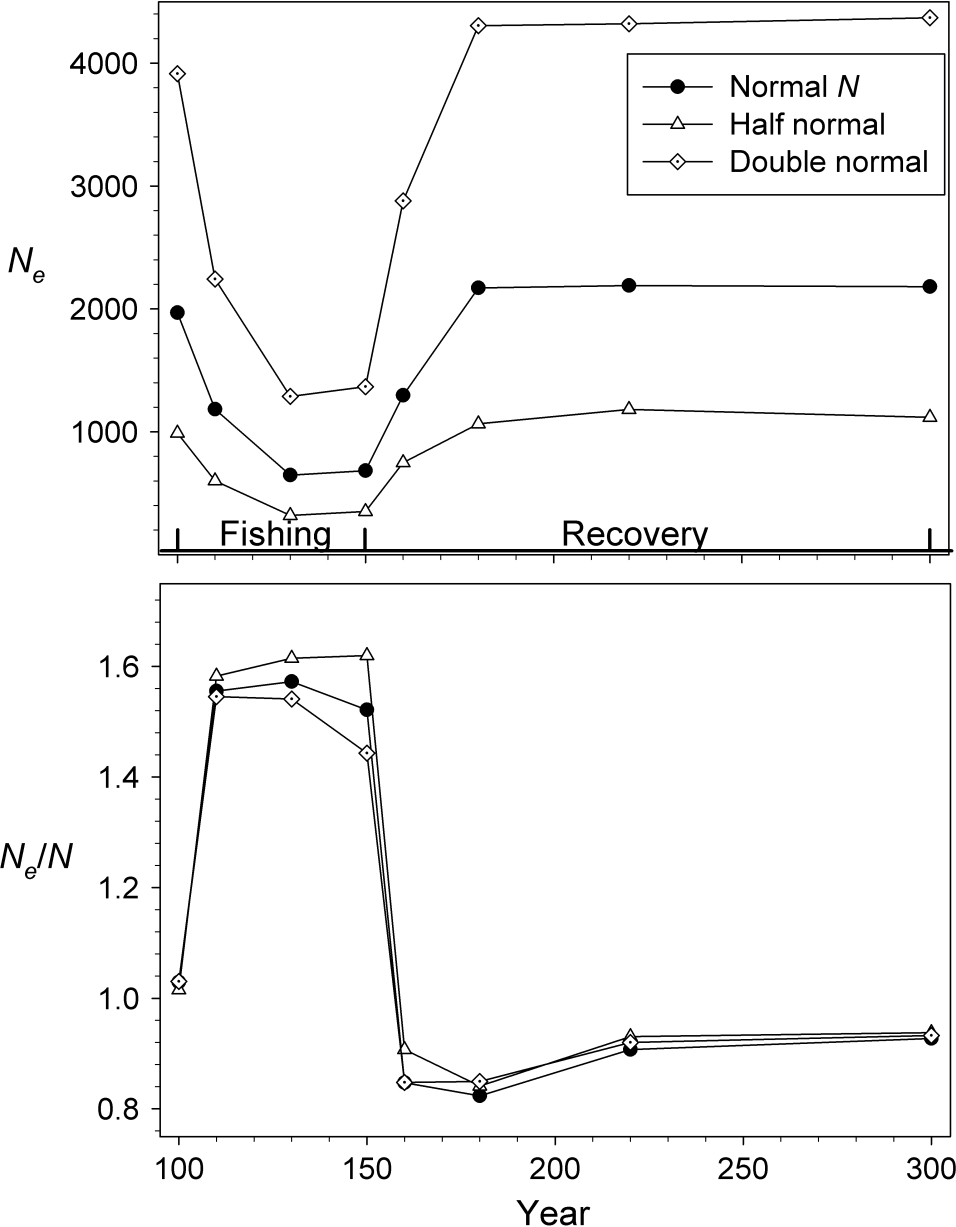
Effects of varying initial population size for simulated cod populations. Results are for selective fishing at *F *=* *0.2 with evolution. The filled circles (Normal *N*) reproduce results for *F *=* *0.2 shown in Fig. 3; the other lines and symbols show results for scenarios in which initial size was half or double the ‘Normal’ level.

*N*_e_ always declined sharply (by 50% or more) during fishing, while the ratio *N*_e_/*N* always increased over the same time period (Fig. 3). This figure shows results for selective and nonselective harvest at *F *=* *0.2 with and without evolution, but this same general pattern was found in every scenario we examined, including those in which the initial population size was doubled or halved (Fig. 4). During recovery, *N*_e_ and *N*_e_/*N* both approached their original values fairly quickly, and this pattern was also consistent across scenarios.

The increases in *N*_e_/*N* during fishing have a simple explanation: declines in *N*_e_ almost exactly mirrored declines in the number of recruits (*N*_1_), while *N* declined at a faster rate (Fig. 2). As discussed later, the more rapid declines in *N* can be attributed to the fact that, whereas declines in recruitment affect *N* and *N*_e_ to the same extent, truncation of age structure caused by increased adult mortality also reduces adult *N* but by itself does not directly change *N*_e_.

#### Effects of evolution

In our model, evolution could increase the probability of maturing at an earlier age through its effect on von Bertalanffy parameters, but in our simulations no individuals matured before age *α* = 3. By the end of fishing at year 150 in scenarios that allowed evolution, the fraction that were mature at young ages (3–5) was 3–14 ×  higher than in the equilibrium population before fishing, compared to 3–10 ×  higher for scenarios that did not allow evolution (Table 2). Thus, most of the age‐structure changes by year 150 can be attributed directly to demographic consequences of increased adult mortality, although evolution enhanced this effect in scenarios where it was allowed. By year 300 (late recovery), the fraction mature at earlier ages was still elevated in scenarios that allowed evolution.

These demographic patterns were reflected in patterns of change in effective population size. Whether or not evolution was allowed had little effect on *N*_e_ and relatively minor effect (±about 15%) on *N*_e_/*N* during harvest. During recovery, however, *N*_e_ and *N*_e_/*N* were both slower to return to their prefishery equilibrium values in scenarios involving evolution, and even at year 300 they had not fully recovered.

#### Selective versus nonselective harvest

Nonselective harvest resulted in more dramatic reductions in overall population size and hence *N*_e_. For example, by the end of fishing (year 150) with *F *=* *0.2 and evolution, size‐selective harvest had reduced *N*_e_ from 1969 to 683, a decline of 65%, while nonselective harvest reduced *N*_e_ from 1959 to 131, a decline of 93% (Fig. 3). These stronger declines in *N* occurred because selective harvest could remove 20% of the biomass by harvesting a relatively small number of larger, older fish, while nonselective harvest that included many smaller fish would have to remove more individuals to take the same biomass. Whether harvest was selective or not had only modest effects on *N*_e_/*N* because additional reductions associated with nonselective harvest were similar for *N*_e_ and *N* (Fig. 3).

#### Different levels of harvest

**Figure 5:**
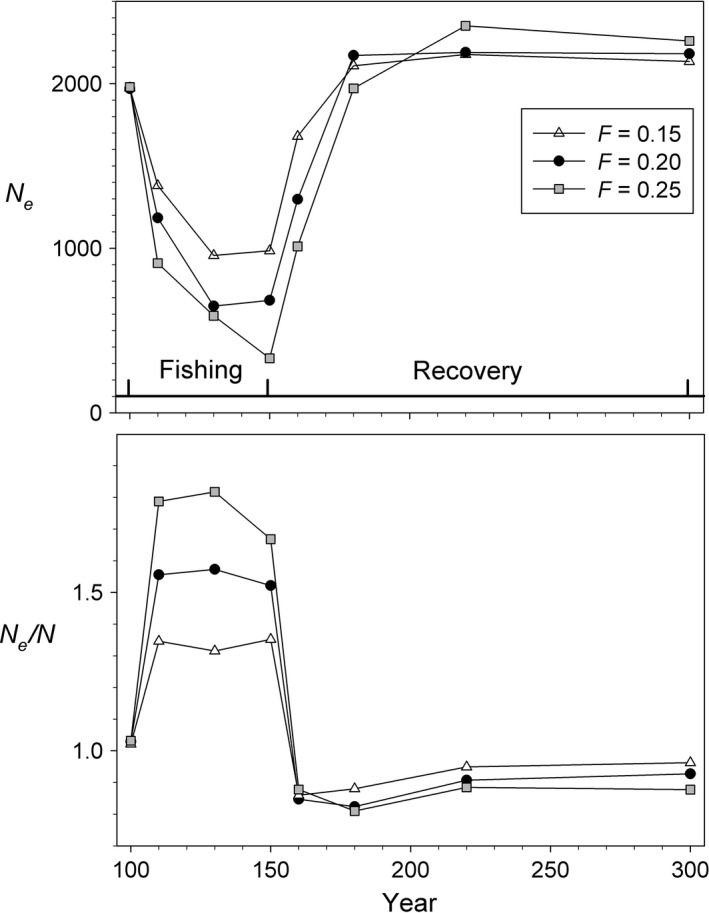
Effects of varying levels of fishing intensity. Results are for simulations with selective fishing with evolution.

Allowing different levels of *F* had predictable consequences for population size and *N*_e_ but did not change the basic patterns described above. Harvesting at a level of *F *=* *0.25 led to greater reductions in *N*_e_, while reducing *F* to 0.15 produced a smaller reduction (Fig. 5). By the end of fishing (year 150), selective harvest at *F *=* *0.25 with evolution had increased *N*_e_/*N* to 1.67, compared to 1.52 and 1.35 for *F *=* *0.2 and 0.15, respectively. All of these patterns related to varying levels of *F* were qualitatively similar under scenarios without evolution (Figure S2).

#### Changes in *T* and *V*_*k•*_

**Figure 6:**
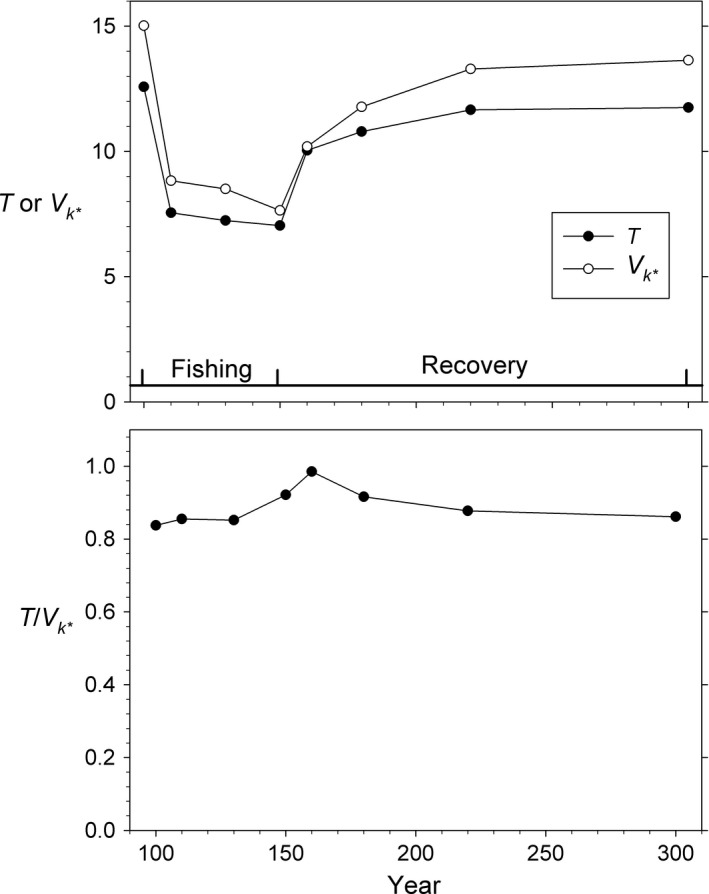
Changes over the course of the simulations in generation length (*T*), lifetime variance in reproductive success (*V*_*k*•_), and their ratio. Results are for selective fishing at *F *=* *0.2 with evolution.

Additional mortality associated with harvest sharply reduced both generation length and lifetime variance in reproductive success, but *V*_*k*•_ declined more rapidly so the ratio *T*/*V*_*k*•_ increased (Fig. 6). When fishing stopped, both *T* and *V*_*k*•_ ncreased again and approached their preharvest equilibrium values, with a predictable lag for scenarios involving evolution. Immediately after fishing stopped, *T* increased more rapidly than *V*_*k*•_, leading to the spike in *T*/*V*_*k*•_ at year 160. Figure 6 shows results for selective fishing with *F *=* *0.2 and allowing evolution, but again this general pattern was evident in all scenarios.

### Analysis of alternative life table

Artificially reducing adult survival from 0.82/year to 0.62/year in cod life table #2 dramatically reduced (from 26% to 4%) the fraction of the adult population made up of individuals age 13 or older, and the population became increasingly dominated by younger individuals (49% of the adult population was age‐7 individuals with annual survival = 0.52, compared to 19% in the real population with natural survival = 0.82; Table 3). Truncating the age structure as adult mortality increased from *d *=* *0.18 to 0.48 reduced the adult population size by 60.8% (Table 4). This is close to the value predicted from eqn (2) (*N*_2_/*N*_1_ = (1/0.48)/(1/0.18) = 0.375, a decline of 62.5%), which would apply to a population with arbitrarily long life span.

**Table 3:** Fraction of adult population in each age class for a Northern cod population experiencing various hypothetical levels of annual adult mortalit

Age class	Adult survival			
	0.82	0.72	0.62	0.52
7	0.192	0.291	0.388	0.490
8	0.157	0.209	0.241	0.255
9	0.129	0.151	0.149	0.132
10	0.106	0.109	0.093	0.069
11	0.087	0.078	0.057	0.036
12	0.071	0.056	0.036	0.019
13+	0.258	0.106	0.036	–*

The first column shows data for the reference population (cod life table #2, for which annual adult survival = 0.82) from Hutchings (2011), as modified by Waples et al. (2013). The other columns depict results for hypothetical populations with the same age‐specific fecundity relationship but different rates of adult survival that reflect natural mortality + fishing mortality.

*In this scenario, maximum age was truncated to *ω* = 12 based on the rules described in the text.

**Table 4:** Results of artificial manipulation of cod life table #2

Adult Mortality (*d*)	Adult *N*	%	*N*_e_	%	*N*_e_/*N*	%	*T*	%	*V*_*k*•_	%	*T*/*V*_*k*•_	%
Scenario I: constant *b*_*x*_; *ϕ* fixed at 3												
0.18	10421	–	8224	–	0.789	–	10.63	–	8.34	–	1.275	–
0.28	6875	−34.0	7172	−12.8	1.043	32.2	9.18	−13.6	8.24	−1.2	1.114	−12.6
0.38	5148	−50.6	6716	−18.3	1.305	65.3	8.45	−20.5	8.07	−3.2	1.047	−17.8
0.48	4084	−60.8	6548	−20.4	1.603	103.2	7.96	−25.1	7.73	−7.3	1.030	−19.2
Scenario II: *b*_*x*_ increases with age; *ϕ* fixed at 3												
0.18	10421	–	7310	−	0.701	–	12.87	–	12.08	–	1.065	–
0.28	6875	−34.0	6361	−13.0	0.925	31.9	10.22	−20.6	10.85	−10.2	0.942	−11.6
0.38	5148	−50.6	6027	−17.6	1.171	66.9	9.02	−29.9	9.97	−17.5	0.905	−15.1
0.48	4084	−60.8	6040	−17.4	1.479	110.8	8.26	−35.8	8.94	−26.0	0.924	−13.3
Scenario III: *b*_*x*_ and *ϕ* increases with age												
0.18	10421	–	8453	–	0.811	–	12.87	–	10.18	–	1.264	–
0.28	6875	−34.0	8252	−2.4	1.200	48.0	10.22	−20.6	7.91	−22.3	1.292	2.2
0.38	5148	−50.6	8431	−0.3	1.638	101.9	9.02	−29.9	6.55	−35.7	1.377	8.9
0.48	4084	−60.8	9083	7.5	2.224	174.2	8.26	−35.8	5.28	−48.1	1.564	23.7

The original life table (Scenario II) had constant adult mortality of *d *=* *0.18 (see Table 3) and fecundity (*b*_*x*_) that increases with age; we also assumed that *ϕ *= 3 for all ages. We considered how increases in adult mortality in this life table would affect key demographic parameters. We also considered two other hypothetical scenarios: one with constant fecundity and *ϕ* fixed at 3 (Scenario I), and one in which fecundity and *ϕ* both increase with age (Scenario III). Results were calculated using AgeNe assuming that the number of recruits produced per year was constant at *N*_*α*_ = 2000. Within each scenario, ‘%’ indicates the percent change from the value when *d *=* *0.18.

In the base population (Scenario II in Table 4), in which fecundity increased with age and *ϕ* was constant, generation length also decreased but by a smaller amount (35.8% for *d *=* *0.48). Although both *N* and *T* are inversely related to adult mortality (Figures S3 and S4; see also Nunney 1991), *T* cannot be lower than the age at maturity (*α *= 7 in this population), and this constrained the rate at which (and amount by which) *T* could be reduced as *d* increased. Furthermore, increasing mortality also reduced *V*_*k*•_ (by 26%), and this largely offset reductions in *N*_e_ caused by lower *T*. As a consequence, *N*_e_ only declined by 17.4% when *d* increased to 0.48. Because this was much less than the reduction in *N*, the ratio *N*_e_/*N* more than doubled, from 0.70 to 1.48.

In Scenario I in Table 4 with constant fecundity (as might be applicable for some harvested species, such as birds), the reduction in *N*_e_ was slightly greater (20.4%). This occurred because under constant fecundity, and starting from relatively high survival, *T* declines more rapidly with increasing mortality than does *V*_*k*•_ (Figure S4).

A different pattern was seen in Scenario III, in which both fecundity and *ϕ* were proportional to age. In this case, increasing mortality had a stronger effect on reducing *V*_*k*•_, such that the ratio *T*/*V*_*k*•_ increased by 23.7% as *d* increased to 0.48; as a consequence, *N*_e_ actually was 7.5% higher with *d *=* *0.48 than with *d *=* *0.18 (again, under the assumption that *N*_*α*_ remained constant).

Results in Table 4 help to illustrate how changes in recruitment and adult mortality interact to determine adult census size [eqn (2)]. For cod life table #2, we lacked empirical data regarding density dependence, so we adopted a simple assumption of no change in recruitment, which would occur only under full productivity compensation (i.e., if the reduced number of adults still produced the same number of offspring per time period). Therefore, this table probably underestimates the reduction in effective size, because *N*_e_ is also linearly related to the number of recruits [eqn (1)]. If instead we had assumed that per capita production of recruits remained constant when mortality increased (i.e., no productivity compensation), then *N*_e_ would have been reduced by an additional 60.8% for *d *=* *0.48. In that event, however, *N* also would have experienced the same additional reduction, so assumptions about density dependence and recruitment had no effect on the *N*_e_/*N* ratio.

In the simulated populations using cod life table #1 (which included density dependence), recruitment dropped substantially with harvest, but not as much as did adult abundance (Fig. 2). This shows at least partial productivity compensation at low density, even if it was not sufficient to fully offset the reduction in adult numbers. It is important to note here that the recruitment and mortality terms in eqns (1) and (2) can interact over time in a feedback loop that can produce cumulative changes over time much larger than predicted from a single iteration. For example, if increased mortality in time period 1 reduces adult *N* and this reduces recruitment, adult *N* will be reduced further in the next time period, and, in the absence of strong productivity compensation, this process can continue until the population collapses. Given our initial conditions, the duration of fishing, and the empirically based form of density dependence we modeled, we found that was the case for simulated populations with *F* greater than about 0.25.

## Discussion

The major results from our study can be summarized as follows:

1. Increasing adult mortality through harvest reduces both census and effective size, but the ratio *N*_e_/*N* increases because *N* is reduced more than *N*_e_.
2. This general result occurs regardless whether harvest is size‐selective or not, and regardless whether evolution of life‐history traits is allowed or not—that is, those other factors affect the outcome in a quantitative way but do not change the qualitative patterns.
3. The intensity of fishing affects the magnitude of change in a predictable way but also does not change these general patterns.
4. The effects of evolution were more pronounced late in the recovery period than they were during harvest. In scenarios without evolution, population parameters rapidly returned to near their equilibrium values after harvest ended, but in the scenarios with evolution the population never achieved its original status by year 300. This was true of biomass, size, age at maturity (Fig. 1), census size and effective size (Fig. 2), and generation length and variance in reproductive success (Fig. 6). Although both *N* and *N*_e_ were higher at year 300 than they were at equilibrium in scenarios with evolution, the proportional increase in *N* was larger, so the *N*_e_/*N* ratio was lower (Figs 2 and 3).

Below we discuss these points and explain why we believe they are not specific to our study system but instead represent quite general expectations for the consequences of increased adult mortality.

### The *N*_e_/*N* ratio

The increase in *N*_e_/*N* during fishing while *N*_e_ went down can be easily understood based on two key insights from inspection of eqns (1), (2), (3). First, both *N* and *N*_e_ are linear functions of the number of recruits that reach age at maturity (*N*_*α*_). This means that any changes in recruitment have proportional changes in *N*_e_ and *N* that are exactly the same, so the ratio *N*_e_/*N* is not affected by recruitment. Therefore, changes in the *N*_e_/*N* ratio are entirely determined by differences in the way *N* and *N*_e_ respond to changes in adult mortality (*d*). The effects of changes in *d* on *N* are again straightforward: increased mortality truncates the age structure and reduces the number of adults as a simple function of the mortality profile as described in eqns (1) and (2). In contrast, *N*_e_ is not directly affected by changes in mortality; it is only indirectly influenced by the effects of changes in mortality on generation length and lifetime variance in reproductive success [eqn (3)].

As discussed above and illustrated in Figure S3, the exact patterns of change in *T* and *V*_*k*•_ associated with a change in adult mortality are complex and depend on age‐specific vital rates and age‐specific *ϕ*. However, because a) the direction of change in *T* and *V*_*k*•_ with increasing mortality is the same (Figure S3 and S4), and b) *T* occurs in the numerator of eqn (3) while *V*_*k*•_ occurs in the denominator, mortality‐mediated changes in *T* and *V*_*k*•_ largely cancel each other (Fig. 6), which greatly constrains the degree to which changes in adult mortality directly affect *N*_e_. To a first approximation, therefore, change in *N*_e_ associated with fishing can be explained solely by changes in recruitment, while changes in *N* depend on both recruitment and mortality. The net result is that increases in adult mortality reduce *N* more than *N*_e_, so the ratio *N*_e_/*N* goes up, even though *N*_e_ will generally decline (absent complete productivity compensation).

Another type of compensation, sometimes termed ‘genetic compensation’, can affect both *N*_e_ and *N*_e_/*N*; this occurs when variance in reproductive success declines at low density, presumably because reduced competition for mates allows otherwise inferior individuals to successfully reproduce. As a consequence of reduced *V*_*k*•_, the ratio *N*_e_/*N* is often higher when population abundance is reduced. Empirical studies that have reported this type of result include Palstra and Ruzzante (2008), Beebee (2009), and Saarinen et al. (2010). Although this could potentially be an important phenomenon in populations subjected to higher adult mortality through harvest, we did not have any empirical information to parameterize this effect with cod. To the extent that it does occur, it would reinforce the pattern we observed in which *N*_e_/*N* increases with fishing intensity.

The *N*_e_/*N* ratios shown in Figs 3, 4, 5, especially those during harvest, are higher than most reported in the literature (e.g., Frankham 1995, Palstra and Fraser 2012). In general, it has been thought that *N*_e_ must be <*N* in natural populations, but recently it has been shown that this is not necessarily the case for species with overlapping generations, particularly those (like cod) with delayed age at maturity (Waples et al. 2013). However, *N*_e_/*N* in iteroparous species is very sensitive to the variance in reproductive success among individuals of the same age and sex (*ϕ*_x_), and high *N*_e_/*N* ratios are only possible if *ϕ*_x_ is relatively low. In this study, we used empirical data for a captive population to parameterize *ϕ*_x_, and as a result it increased from about one at age at maturity to over four by age 25. Values of *ϕ*_x_ in wild populations could potentially be much higher, especially in species that experience ‘sweepstakes’ reproductive success as proposed by Hedgecock (1994). Unfortunately, however, very few estimates of *ϕ*_x_ are available for wild populations of any species. Nevertheless, it is easy to evaluate how hypothetical values would affect the *N*_e_/*N* ratio for the simulated populations. For example, in our simulated populations based on cod life table #1, initial *N*_e_/*N* would be reduced from nearly 1.0 to below 0.1 if the age‐specific *ϕ*_x_ values we used were all multiplied by a fixed factor 50 (Figure S4A). Such a population would have much lower *N*_e_ and *N*_e_/*N*, but the pattern of change over time in these parameters (Figure S4B) would be similar to that shown in Figs 3, 4, 5.

Finally, because changes in adult mortality can have large effects on the *N*_e_/*N* ratio (as demonstrated here), and because anthropogenic changes to all of earth's ecosystems have dramatically changed mortality profiles for many species, it is risky to assume that the *N*_e_/*N* ratio is constant, absent a good reason to believe that is the case.

### Effects of evolution

The typical evolutionary response to increased adult mortality is to evolve mechanisms that allow earlier maturation, which increases the chances of having at least one opportunity to reproduce before being harvested. What are the likely consequences for *N*_e_? If increased adult mortality causes an evolutionary response toward earlier maturation, that would reduce generation length and, all else being equal, that would reduce *N*_e_ [eqn (1)]. However, earlier maturation could also mean that more total individuals survive to maturity, which would increase the number of recruits (*N*_*α*_) and, all else being equal, increase *N*_e_. Therefore, the net effects of evolution on *N*_e_ and *N*_e_/*N* are expected to depend on the relative importance of these two factors. The effects on generation length are easier to predict, while those on recruitment depend on assumptions about ecological processes such as competition and density dependence.

In the simulated populations, reductions in *N* caused by higher harvest rates enhanced juvenile growth and survival through relaxation of density dependence, and as a consequence, a larger fraction of individuals matured at earlier ages (Table 2). This was a purely ecological phenomenon that also caused age‐structure shifts in populations without evolution. Allowing evolution of age at maturity, therefore, only added a relatively small component to a fundamentally ecological process (compare last two columns in Table 2). This tended to blur the distinction between results for scenarios that did and did not allow evolution, at least during the period of harvest.

The major (and quite consistent) difference between the evolution and nonevolution scenarios can be found at the end of the long recovery period (year 300), by which time the vital rates of all populations simulated without evolution had returned to essentially the same place they were before harvest commenced. In contrast, at year 300 in scenarios that involved evolution, *N*_e_ was always slightly higher and *N*_e_/*N* slightly lower than it was in the equilibrium preharvest population. This result is consistent with empirical observations from other studies (e.g., Pigeon et al. 2016) that document rapid evolution of life history under strong selection, but slower evolution toward initial phenotypes once selection is relaxed, presumably because selection in the wild is seldom as strong as selection humans impose through harvest (Allendorf and Hard 2009).

Two factors combined to produce the higher *N*_e_ at year 300: higher *N* (Fig. 2) and higher *T*/*V*_*k*•_ (Fig. 6) compared to their values at year 100. However, the ratio *N*_e_/*N* was lower at year 300 than at year 100. This occurred because evolution of the age‐at‐maturity reaction norm toward earlier maturity meant that a larger fraction of the population was mature at an earlier age, and this increased adult *N* faster than it did *N*_e_. The net effect was a reduction in *N*_e_/*N*, even though *N*_e_ was slightly higher in late‐recovery populations that allowed evolution than it was at preharvest equilibrium.

### Model assumptions

The Felsenstein–Hill models that AgeNe is based upon assume constant population size and stable age structure. These assumptions were met in the preharvest equilibrium population (year 100) and nearly met in the late recovery phases (after about year 200), but harvest led to rapid changes in population demography that affected data collected in years 110–180. Therefore, because AgeNe calculates *N*_e_ for individual cohorts based on vital rates calculated at specific points in time, our results are best interpreted as estimates of instantaneous *N*_e_ that would apply to a population that remained stable with those mean vital rates. Nevertheless, several lines of evidence suggest that our results should be fairly robust to these demographic changes. Felsenstein (1971) showed that his model accurately estimates *N*_e_ for populations that are increasing or declining at a constant rate, and this was approximately met during the decline due to fishing and the resulting rebound after fishing stopped. Waples et al. (2011, 2014) showed that eqn (1) provides robust results in simulated populations that incorporate random demographic stochasticity and with *N*_e_ as low as 200 (lower than the levels reached in any of our scenarios except those with nonselective fishing). Furthermore, substantial generational overlap and long adult life span (as are found in cod populations) help to buffer a population against cyclical environmental fluctuations (Gaggiotti and Vetter 1999). Finally, although Hill (1972; 289) did not formally evaluate the assumptions of constant population size and random mating, he did provide arguments why he believed that ‘neither effect has much influence on effective population size’.

We did not simulate very small effective sizes (*N*_e_ <100) because that is difficult to do in a long‐lived species with many age classes. If effective size is that small, random changes in allele frequency can overwhelm the effects of selection, which would make predictions regarding FIE less reliable. However, because most of the changes we reported were dominated by demographic changes related to increases in adult mortality rather than evolutionary changes, we believe our results would also be qualitatively true for smaller *N*_e_ values than we modeled.

The AgeNe model also assumes that probabilities of survival and reproduction are independent across time. That will not always be the case. If, for example, individuals (especially females) who reproduce in one time period have a reduced probability of reproducing for one or more subsequent time periods, *N*_e_ will be slightly higher than calculated under AgeNe because skip breeding tends to reduce extreme variation in lifetime reproductive success (Waples and Antao 2014). Conversely, if certain individuals are consistently above or below average in their reproductive output, *N*_e_ will be reduced (Lee et al. 2011). Although these phenomena can influence effective population size, they should not affect the general patterns of change in *N*_e_ and *N*_e_/*N* in response to increases in adult mortality.

### Implications for conservation and management

We demonstrated that *N*_e_ is likely to decline, perhaps substantially, in response to elevated adult mortality associated with harvest. Our results thus support the conclusion by Marty et al. (2015) that failure to account for stochastic processes associated with reduced *N*_e_ can lead to incorrect conclusions about eco‐evolutionary dynamics associated with fishery‐induced evolution. However, these results also add some important nuances to our understanding of this complex topic.

First, the good news is that increasing harvest rates can be expected to increase the *N*_e_/*N* ratio. This means that the proportional reductions in *N*_e_ will be smaller than the effects of harvest on total abundance. As the latter are easier to predict, the expected reduction in *N* can be used as an upper limit to the expected reduction in *N*_e_, with the expectation that increases in the *N*_e_/*N* ratio will at least partially buffer the overall reduction in effective size.

The second important point is that although adding anthropogenic harvest to natural mortality can promote fishery‐induced evolution, direct demographic consequences of elevated adult mortality explain most of the reductions in effective size that we observed in the modeled populations. Reductions in *N*_e_ are caused primarily by reductions in recruitment, as the effects of elevated harvest on *T* and *V*_*k*•_ tend to cancel each other [eqn (3)]. We did, however, find that long after harvest stopped, *N*_e_ was higher in the scenarios that involved evolution, which indicates a potentially important role for evolution in maintaining genetic diversity in populations recovering from periods of elevated harvest‐related mortality.

Although this does not directly relate to effective size, it is worth noting that, because substantial generational overlap and the storage effect (Warner and Chesson 1985) help buffer a long‐lived species against environmental fluctuations, truncation of age structure resulting from increased adult mortality will reduce this buffering capacity, leaving the population more vulnerable to random events.

The eco‐evolutionary patterns described here are quite general and should be applicable to a wide range of species that experience increased mortality from anthropogenic factors, including but not limited to harvest. In a recent study, Dowling et al. (2014) monitored effective size over 15 years in a species (razorback sucker, *Xyrauchen texanus*) experiencing reduced survival in altered habitat and found that effective size was stable or increased while *N* declined, so *N*_e_/*N* increased. These were genetically based estimates and did not consider demography, but the authors also used AgeNe to evaluate the consequences of truncating the life span from 44 to 20 years. Dowling et al. (2014) found this truncation caused little change in *N*_e_/*N*, so they concluded that the increase in the effective: census size ratio was due to reduced variance in reproductive success. However, simply truncating a life table at a certain age does not properly mimic a scenario with increasing adult mortality, as the latter will reduce abundance in all ages from age at maturity onwards. We altered the life table for razorback sucker (published in Waples et al. 2013) by reducing adult survival from 0.8 to 0.6 and truncating at 20 years, and this raised *N*_e_/*N* from about 1.0 to 1.6, comparable to changes we report here. Thus, although it is certainly possible that variance in reproductive success has been reduced in this species, it is not necessary to postulate that to explain the empirical pattern in the estimates of *N*_e_/*N*.

One important factor that applies to species subject to trophy hunting is that harvest that targets males can skew the sex ratio and hence reduce *N*_e_ (Coltman et al. 2003; Hard et al. 2006). Although the AgeNe model can easily incorporate sex‐specific vital rates to fully account for sex‐ratio effects on *N*_e_, harvest of cod is thought to be sex‐neutral and we do not have evidence for sex‐based differences in survivorship. We can, however, predict the general consequences of male‐targeting trophy hunting on *N*_e_ and *N*_e_/*N* using the framework developed here. When males and females have different vital rates, the simple formula developed by Wright (1938) can be used to calculate overall *N*_e_ as a function of the effective numbers of females and males. Sharply increasing mortality of adult males will reduce male *N* but at the same time will increase male *N*_e_/*N*, for reasons described above. As a consequence, male *N*_e_ will not decline as fast as male *N*, so the effects on overall *N*_e_ will be less than would be predicted simply from the reduction in the number of adult males. The net results for overall *N*_e_ will depend on population‐specific patterns in vital rates that determine how the ratio *T*/*V*_*k*•_ changes with increasing adult mortality.

## Supporting information


**Data S1.** Age‐specific variance in reproductive success.
**Figure S1.** Patterns of adult survival (individuals age 3+) as a function of age in simulated cod populations.
**Figure S2.** Effects of varying levels of fishing intensity for simulated cod populations. Results are for selective fishing without evolution (i.e., as in Fig. 5, main text, but without evolution).
**Figure S3**. Theoretical relationship between adult survival (assumed to be constant at annual rate 1–*d*) and generation length (*T*) and lifetime variance in reproductive success (*V*_*k*•_).
**Figure S4**. Effects of increasing *ϕ* on *N*_e_ and the *N*_e_/*N* ratio in simulated cod populations.

**Table S1.** Age‐specific vital rates for simulated cod populations at representative time periods.
**Table S2.** Age‐specific vital rates for cod life table #2 under different assumptions about adult survival.
