# Supplementary material for: Harvest‐induced evolution and effective population size
Source: Evol Appl. 2016 Apr 8;9(5):658–72. doi: 10.1111/eva.12373 (PMC4869408; doi:10.1111/eva.12373)
Supplement: Supplementary file 1 — Data S1. Age‐specific variance in reproductive success. Figure S1. Patterns of adult survival (individuals age 3+) as a function of age in simulated cod populations. Figure S2. Effects of varying levels of fishing intensity for simulated cod populations. Results are for selective fishing without evolution (i.e., as in Fig. 5, main text, but without evolution). Figure S3. Theoretical relationship between adult survival (assumed to be constant at annual rate 1–d) and generation length (T) and lifetime variance in reproductive success (V k•). Figure S4. Effects of increasing ϕ on N e and the N e/N ratio in simulated cod populations. [file EVA-9-658-s001.pdf]

Harvest-induced evolution and effective population size

Anna Kuparinen, Jeffrey A. Hutchings, and Robin S. Waples

Supporting information

See separate Excel file for vital rates for selected time periods for simulated data (Table S1) and for artificial life tables for Newfoundland cod (Table S2).

Age-specific variance in reproductive success

To develop age-specific estimates of $\phi = V_k/\bar{k}$ = the ratio of variance to mean reproductive success over one time period for individuals of the same age and sex, we drew on experimental data for three captive populations in which parentage analysis was used to assign offspring (fertilized eggs) to potential male parents (Rowe et al. 2008). In each spawning event for each population, the length of each parent and the number of assigned offspring was recorded, and parental lengths were binned into assumed ages based on age-size relationships for Northern cod described by Hutchings (1999). For each age in each population with data for at least 5 individuals, we calculated $\bar{k}$ and the raw, unbiased sample estimate of V_k. AgeNe calculations require that $V_k/\bar{k}$ be scaled to the actual, stable-population-size fecundity for that age and sex, which we obtained from AgeNe output. We rescaled V_k to the target $\bar{k}$ using a method first proposed by Crow and Morton (1955) and implemented by Waples (2002, equation 3). We then averaged the scaled ϕ ratios across populations to obtain overall estimates of ϕ for ages 3-8. These data show that estimated, scaled ϕ was close to 1 for age 3 and increased in a roughly linear fashion through age 8, when scaled $\phi = 1.83$. Ignoring an outlier at age 7, linear regression of the other datapoints produced the following relationship: scaled ϕ at age $x = 0.451 + 0.164x$ (adjusted $R^2 = 0.94$). We used this equation to estimate ϕ for ages older than 8.

We also adjusted ϕ for each age to account for the fact that not all individuals of a given age were mature. The data collected by Rowe et al. (2008) are relevant to variance in reproductive success among adults. For AgeNe calculations, however, we need to know the mean and variance in reproductive success among all individuals of a given age, mature or not. At each age for which the fraction mature was not 0 or 1, we had two groups of individuals—those that were mature, which had ϕ as described above, and those that were immature, which produced no offspring. We then calculated an adjusted, age-specific ϕ based on the fraction of individuals mature at each age as recorded in the simulations (Table S1).

Crow, J.F., and N.E. Morton 1955. Measurement of gene frequency drift in small populations. Evolution 9:202–214.

Hutchings, J.A. 1999. The influence of growth and survival costs of reproduction on Atlantic cod, *Gadus morhua,* population growth rate. *Canadian Journal of Fisheries and Aquatic Sciences* 56: 1612-1623.

Rowe, S., Hutchings, J.A., Skjæraasen, J.E., and L. Bezanson. 2008. Morphological and behavioural correlates of reproductive success in Atlantic cod *Gadus morhua*. *Mar. Ecol. Prog. Ser.* 354: 257-265.

Waples, R.S 2002. Evaluating the effect of stage-specific survivorship on the N_e/N ratio. Molecular Ecology 11:1029-1037.

(see main text for other references)

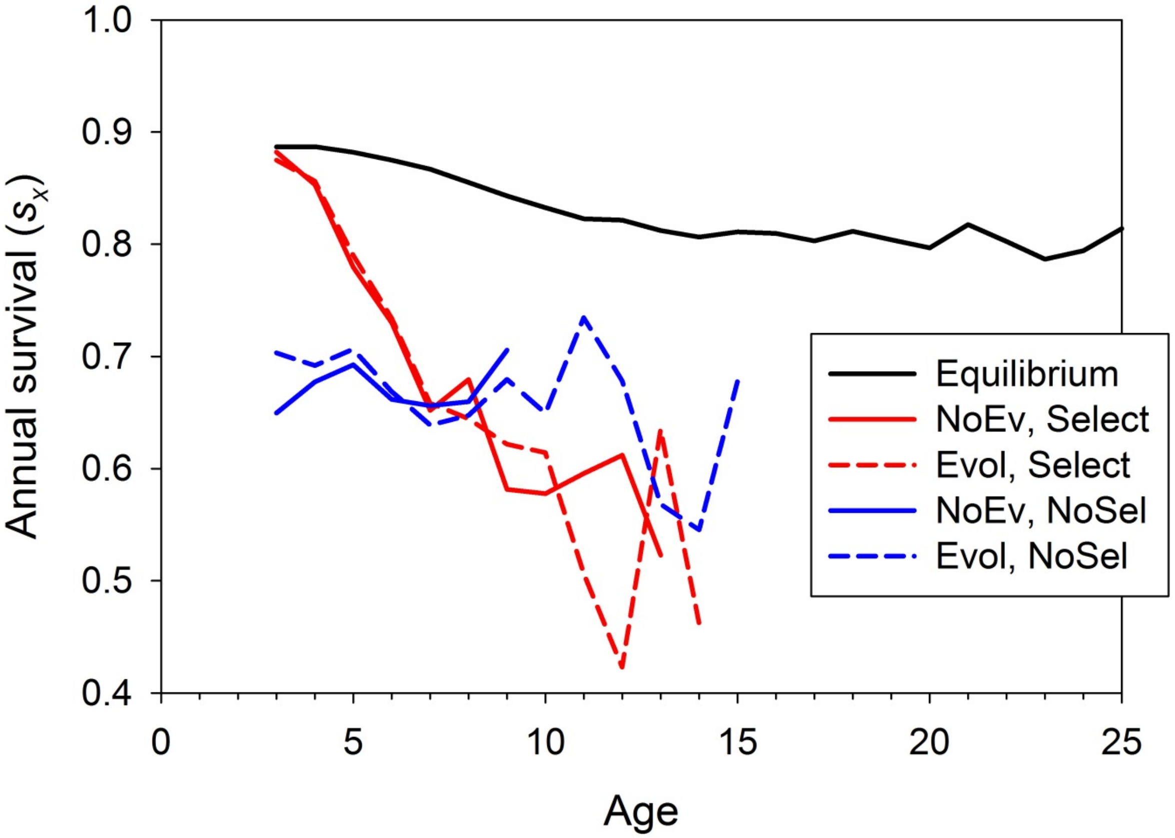

Figure S1. Patterns of adult survival (individuals age 3+) as a function of age in simulated cod populations. The black line is equilibrium conditions before harvest begins (year 100); colored lines represe0nt conditions at the end of fishing (year 150). Results are shown for scenarios with and without evolution and with and without selective harvest; in all cases the level of fishing was $F = 0.2$. In data for year 150, the life tables were truncated when insufficient numbers of individuals survived to compute vital rates.

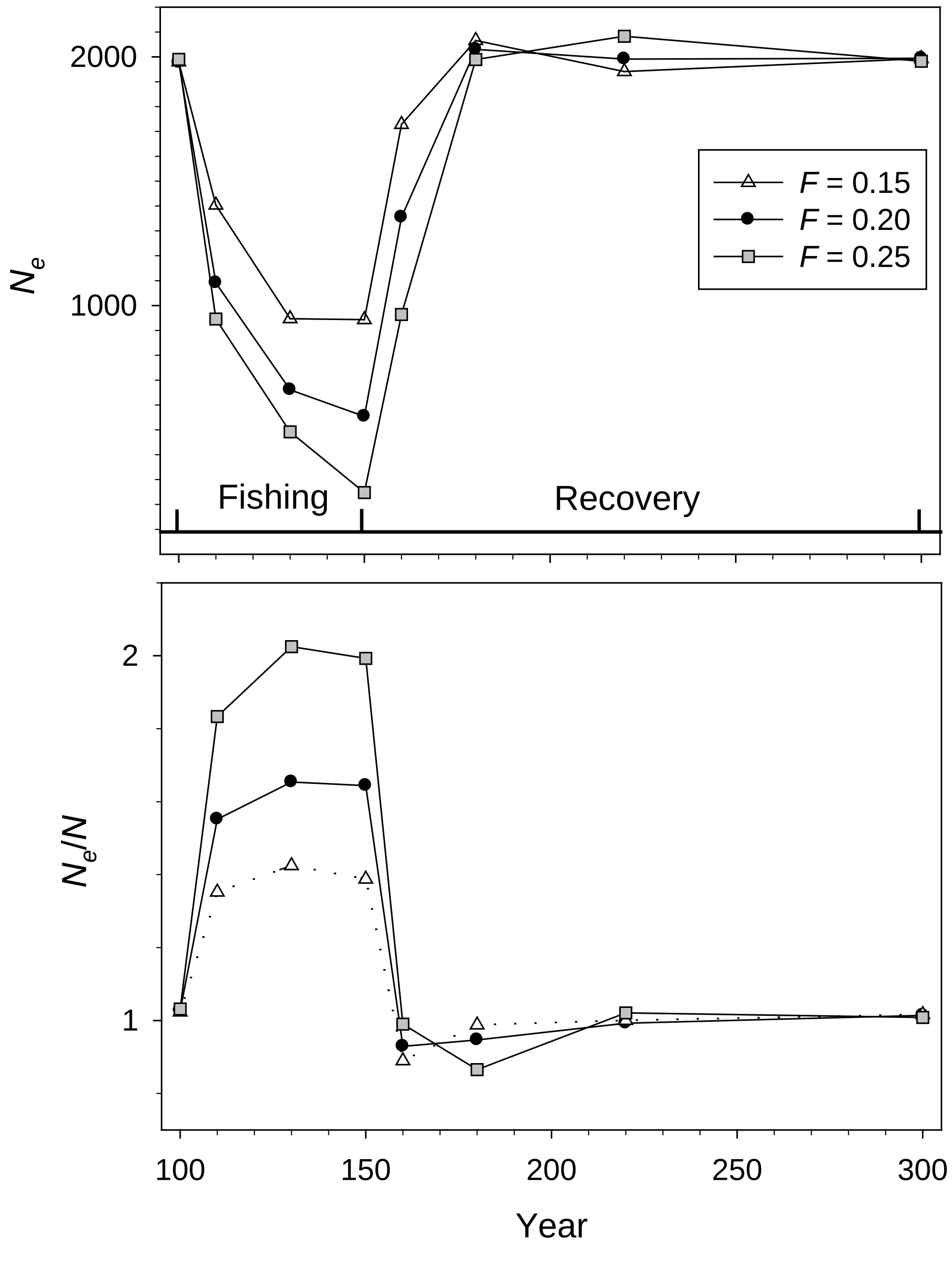

Figure S2. Effects of varying levels of fishing intensity for simulated cod populations. Results are for selective fishing without evolution (i.e., as in Fig. 5, main text, but without evolution).

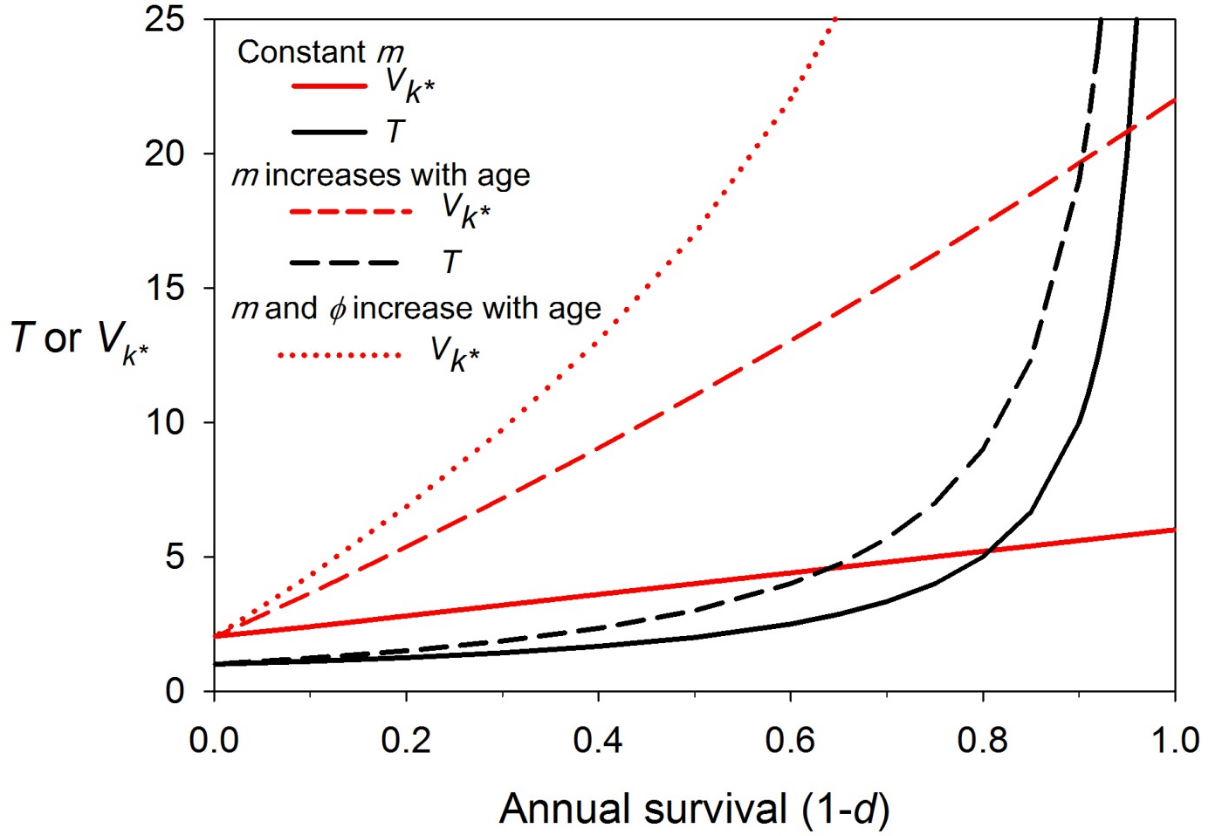

Figure S3. Theoretical relationship between adult survival (assumed to be constant at annual rate 1–*d*) and generation length (*T*) and lifetime variance in reproductive success ($V_{k\bullet}$). These are analytical and numerical results based on relationships described in Waples (accepted pending revision). Three scenarios are considered: constant fecundity (*m*) with $\phi = 1$; fecundity that is proportional to age ($m_x = \beta x$) with $\phi = 1$; and m_x and ϕ both proportional to age. Changes in ϕ do not affect generation length, so in the last scenario the curve for *T* vs 1-*d* is also given by the black dashed line.

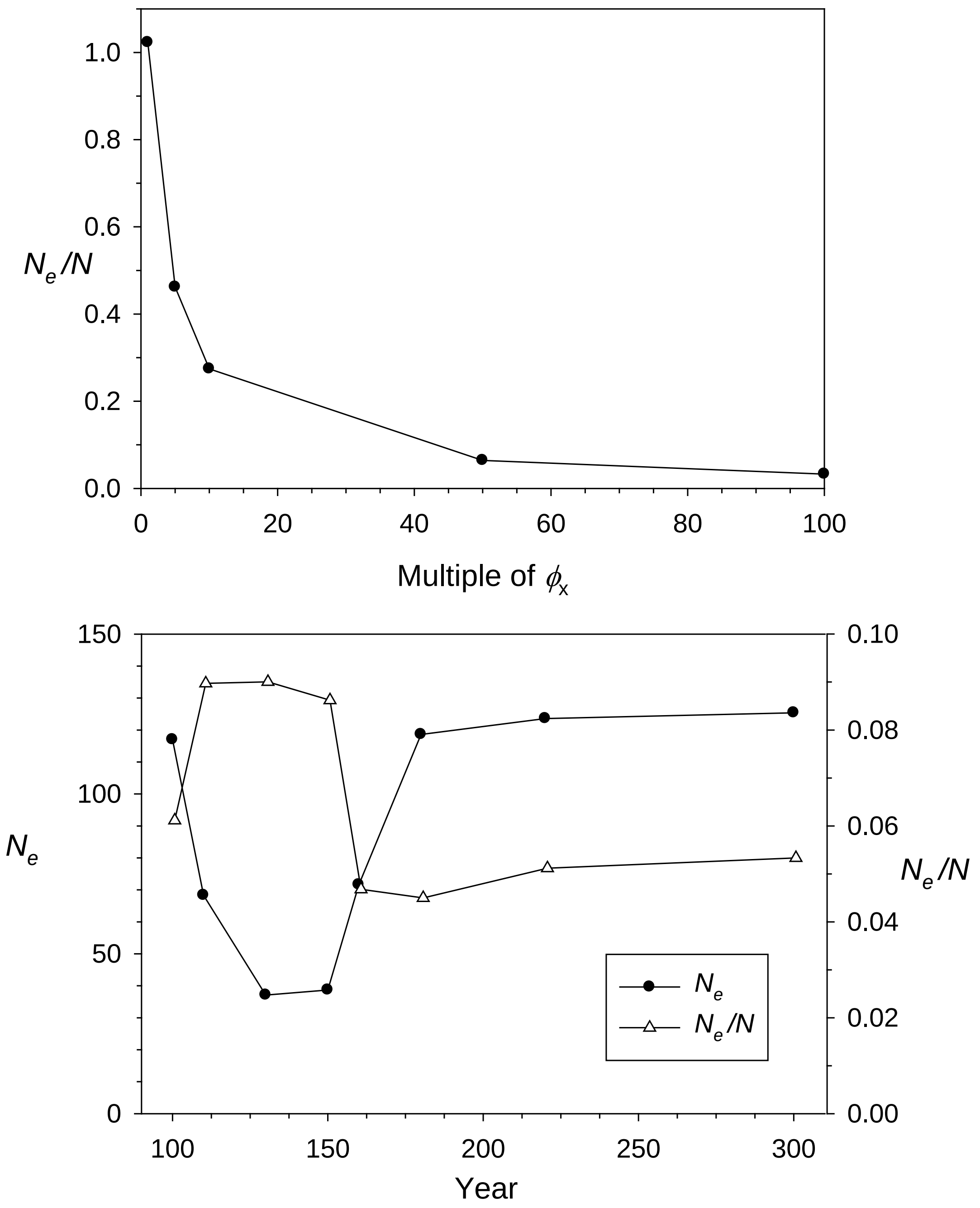

Figure S4. Effects of increasing ϕ on N_e and the N_e/N ratio in simulated cod populations. Top: Effects on N_e/N if ϕ_x for each age is multiplied by a constant factor. These results apply to the equilibrium population at year 100; the reference dataset for the initial datapoint (multiplier = 1) is given in column F, rows 3-27, of Table S1. Bottom: Resulting N_e and N_e/N if ϕ_x for each age is multiplied by 50. These results used the life tables for the simulations with selective fishing at F = 0.2, with evolution.
